# BALL-SNP: combining genetic and structural information to identify candidate non-synonymous single nucleotide polymorphisms

**DOI:** 10.1186/s13073-015-0190-y

**Published:** 2015-07-01

**Authors:** Sabine C. Mueller, Christina Backes, Olga V. Kalinina, Benjamin Meder, Daniel Stöckel, Hans-Peter Lenhof, Eckart Meese, Andreas Keller

**Affiliations:** Chair for Clinical Bioinformatics, Saarland University, Saarbrücken, Germany; Department of Internal Medicine III, University Heidelberg, Heidelberg, Germany; DZHK (German Centre for Cardiovascular Research), Berlin, Germany; Center for Bioinformatics Saar, Saarland University, Saarbrücken, Germany; Department of Human Genetics, Saarland University, Saarbrücken, Germany; Max Planck Institute for Informatics, Saarbrücken, Germany

## Abstract

**Background:**

High-throughput genetic testing is increasingly applied in clinics. Next-Generation Sequencing (NGS) data analysis however still remains a great challenge. The interpretation of pathogenicity of single variants or combinations of variants is crucial to provide accurate diagnostic information or guide therapies.

**Methods:**

To facilitate the interpretation of variants and the selection of candidate non-synonymous polymorphisms (nsSNPs) for further clinical studies, we developed BALL-SNP. Starting from genetic variants in variant call format (VCF) files or tabular input, our tool, first, visualizes the three-dimensional (3D) structure of the respective proteins from the Protein Data Bank (PDB) and highlights mutated residues, automatically. Second, a hierarchical bottom up clustering on the nsSNPs within the 3D structure is performed to identify nsSNPs, which are close to each other. The modular and flexible implementation allows for straightforward integration of different databases for pathogenic and benign variants, but also enables the integration of pathogenicity prediction tools. The collected background information of all variants is presented below the 3D structure in an easily interpretable table format.

**Results:**

First, we integrated different data resources into BALL-SNP, including databases containing information on genetic variants such as ClinVar or HUMSAVAR; third party tools that predict stability or pathogenicity *in silico* such as I-Mutant2.0; and additional information derived from the 3D structure such as a prediction of binding pockets. We then explored the applicability of BALL-SNP on the example of patients suffering from cardiomyopathies. Here, the analysis highlighted accumulation of variations in the genes *JUP*, *VCL*, and *SMYD2*.

**Conclusion:**

Software solutions for analyzing high-throughput genomics data are important to support diagnosis and therapy selection. Our tool BALL-SNP, which is freely available at http://www.ccb.uni-saarland.de/BALL-SNP, combines genetic information with an easily interpretable and interactive, graphical representation of amino acid changes in proteins. Thereby relevant information from databases and computational tools is presented. Beyond this, proximity to functional sites or accumulations of mutations with a potential collective effect can be discovered.

## Background

The study of non-synonymous polymorphisms (nsSNPs) as genetic factors in human diseases and their contribution to phenotypic traits is essential in human healthcare. Although only a small fraction of genetic variations cause nsSNPs, over 85 % of such mutations are associated with a specific disease [1]. NsSNPs can introduce premature stop codons, consequently producing functionally incompetent truncated proteins, and hence, are possibly lethal. Viable nsSNPs frequently result in a single amino acid change within a protein sequence and, thus, can alter protein function comprising folding, stability, and binding of other proteins or ligands.

The increasing adoption of Next-Generation Sequencing (NGS) in clinical applications leads to a substantial amount of novel nsSNPs. Since the experimental analysis to gain knowledge concerning the pathogenicity of these is laborious and time-consuming, computational approaches have been developed to predict the impact of an amino acid substitution on protein function *in silico* [2, 3]. Most of the existing computational approaches predict the pathogenic effect using statistical methods, machine learning techniques, or protein evolution models, based on features such as sequence homology, biochemical properties, and structural information (hydrogen-bond network, solvent accessibility, and so on). Besides, there are computational methods based on potential energy functions, force fields, and molecular dynamics, which analyze the change in a protein’s stability, dynamics, and interactions to consequently derive the impact of an amino acid substitution [4, 5]. These methods, however, can also be time-consuming and are generally used for small-scale investigations [6].

Beyond the influence of single mutations, we started to study the influence of several nsSNPs in the same protein, which may have a cumulative effect [7, 8]. From a medical point of view, especially the individual combination of nsSNPs may play a unique and crucial role in clinical diagnostics, in particular, within the context of complex genetic disorders. Since a protein’s structure, dynamics, and interactions are interrelated, nsSNPs may change several properties of a protein simultaneously [9]. Besides many tumor entities, cardiovascular disorders such as cardiomyopathies are known to be influenced by numerous genetic variations and additional environmental circumstances [10]. Thus, computational approaches able to assess synergetic effects of multiple nsSNPs in one single protein or in a complex of interacting proteins are being currently developed.

To capture the relationship of multiple nsSNPs with disease phenotypes, besides sequence features, structural information is essential [7, 11]. The analysis of a collective effect of several mutations within one protein requires information on their three-dimensional (3D) environment and interaction. Are they located close to each other? Do they change the hydrogen bond network stabilizing the protein? Do they alter hydrophobicity and charge steering a protein’s folding? Moreover, the 3D location of substituted amino acids shed light on the implied impact on the protein’s function: An exchange of buried residues may, for example, affect protein stability and folding, whereas mutations located on the protein surface, close to putative binding sites can alter binding affinities.

To address this, we developed BALL-SNP, a novel tool based on the Biochemical Algorithms Library (BALL) [12], a molecular modeling framework, which provides robust and sophisticated algorithms on structural bioinformatics. BALL-SNP enables the assessment of the functional impact of multiple nsSNPs in a single protein by visualizing the mutated residues within a wild type structure, performing a cluster analysis, and supplementing it with the available information on the pathogenicity of the nsSNPs from different databases [13, 14]. Additionally, putative binding pockets in the protein structure, as well as protein stability changes are predicted. Based on the generated information and the 3D visualization, the user can hypothesize whether the amino acid substitutions can produce a collective effect due to mutual interaction or have an influence on binding and stability. In consequence, candidate nsSNPs for further studies can be selected.

## Methods

### Dataset

A valid and high-quality dataset is essential when analyzing the phenotypic effect of nsSNPs on human health. We analyzed a NGS dataset of 639 patients screened for the full sequence of 76 genes, clinically relevant for dilated cardiomyopathy (DCM) [15]. The dataset involves 842 nsSNPs. The sequencing was performed on IlluminaHiSeq instruments. About 99.1 % of the targeted genomic region was covered at least 50-fold. In consequence, the used dataset is of high clinical quality.

According to available annotations in dbSNP [16], SwissProt [17], and the Human Gene Mutation Database (HGMD) [18], the DCM dataset comprises 192 benign-labeled and 147 disease-linked nsSNPs. About 55 % of the data have no available annotation information [7].

### BALL-SNP

**Fig. 1:**
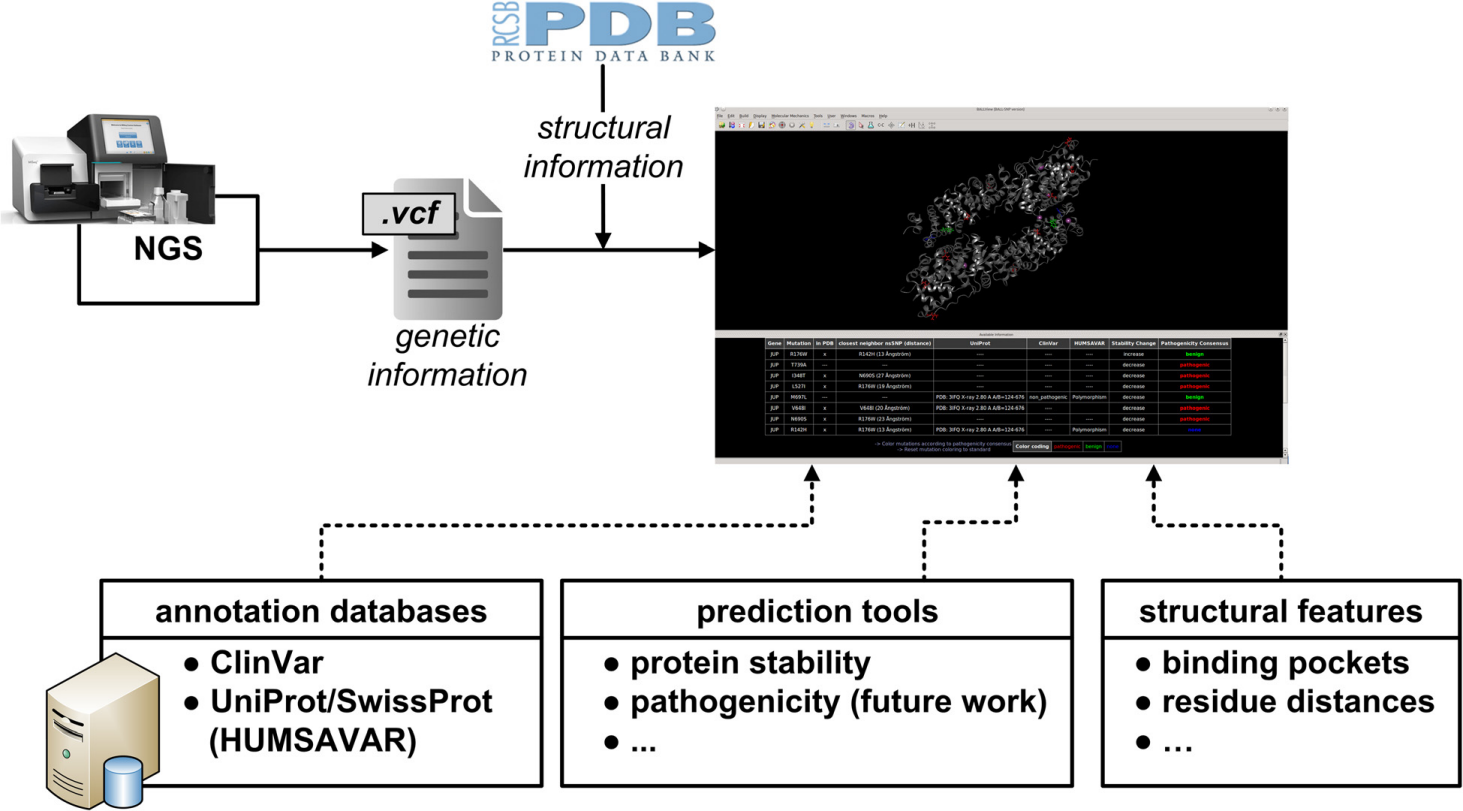
BALL-SNP workflow

We introduced a new pipeline for the assessment of multiple nsSNPs in NGS data. Our tool BALL-SNP is based on the Biochemical Algorithms Library (BALL) [12] and integrated in BALL’s visualization front-end BALLView [19]. BALL is a comprehensive application framework for rapid software prototyping, which offers a large number of molecular data structures and algorithms allowing for sophisticated development of new approaches. Since we aim to combine genetic and structural information, while ensuring intuitive usability, we take advantage of BALL’s rich functionality. We extended the versatile C++ class library by adding functionality to import and process variant call format (VCF)-based file formats used in DNA sequencing and SNP calling. We furthermore embedded the currently most important SNP annotation databases and corresponding parsing methods. In addition, we introduced a first version of a compute server and the associated request functionality allowing for straightforward integration of available prediction tools. However, to be independent of the software maintenance by a third party and to guarantee stable performance, we only focus on the integration of available stand-alone software tools, installed on the created compute server. Figure 1 outlines the BALL-SNP workflow along with all incorporated data sources.

Besides the 3D visualization, we display additional generated information in an accessible HTML-based interface, facilitating a clearly arranged presentation. Moreover, as BALL-SNP is implemented on top of the standard molecular modeling tool BALLView, an intuitive and direct interaction of the user with the visualized 3D structure representations is possible.

## Results and discussion

### Input formats

To ensure straightforward usability, we currently offer two different input formats: an ANNOVAR-based input [20], as well as a simple tab-separated format. Hence, users are enabled to adopt the output obtained from standard SNP calling software such as ANNOVAR without substantial re-formatting, as well as use SNP information from different sources compiled in a simple tab-separated input file. The ANNOVAR version used for testing refers to the version from 12.11.2014. We used the default parameters and set --buildver to hg19.

BALL-SNP focuses on the analysis of the pathogenic relevance of nsSNPs. The SNP calling process, however, may have great influence on the results of the BALL-SNP analysis. In consequence, the user should carefully adopt the SNP calling parameters to his application purpose.

Since 3D structure information is essential for the analysis of nsSNPs accumulated within one single protein, we automatically extract the PDB identifier of the largest available 3D structure from UniProt [14]. The chosen PDB structure, then is automatically loaded from the Protein Data Bank (PDB) [21]. To maintain flexibility, we also provide the possibility to state a preferred PDB identifier within both input formats or to specify a file name with a user-built 3D model of the query protein. Further input formats can easily be added.

### Pathogenicity information

Experimentally gained knowledge about nsSNPs is deposited and curated in different databases. Some of these databases provide additional information concerning the pathogenicity and clinical significance of a nsSNP. To make use of this knowledge, we include information from SwissProt/UniProt [14] and ClinVar [13] within BALL-SNP. In particular, SwissProt/UniProt collects human polymorphisms and disease mutations (annotated in the HUMSAVAR document) assigned according to literature reports on probable disease association. ClinVar is based on the dbSNP [16] and reports human variations and interpretations of the relationship of these variations to human health by providing clinical significance information.

Currently, we are focusing on selected important databases that report nsSNP pathogenicity. The embedded database module, however, can easily be extended to include further databases and annotation sources.

### Predicting binding pockets

In addition to known information on pathogenicity from databases and *in silico* prediction, further information may provide clinicians essential input. Among these, the proximity of nsSNPs to functional sites such as binding pockets for ligands plays a crucial role. BALL-SNP predicts active sites, which often are located in the largest surface cleft, based on the Putative Active Sites with Spheres (PASS) method [22], that use probe spheres to characterize regions of buried volume on a protein surface. Based on size, shape, and burial extent of these volumes, positions, which putatively represent binding sites, are identified. The predicted active sites are visualized as spheres in BALL-SNP, which represent their centers.

### Protein stability change

Proteins properly folded have minimal potential energy and are usually stable. Amino acid substitutions introducing a change in the protein sequence can have a significant impact on the potential energy of the protein structure, and thus its folding and stability. Consequently, the analysis to which extent a mutation affects protein stability with respect to the wild type, extends the understanding of the mutation impact on protein function and the genotype-phenotype relationship, accordingly.

Several methods to predict the change of a protein’s binding free energy exist [5, 23]. Since I-Mutant was shown to have better performance compared to other tools [24], we set up a compute server running freely available I-Mutant 2.0 code [25]. I-Mutant 2.0 automatically predicts protein stability changes caused by single point mutations in protein sequence using support vector machines (SVMs). BALL-SNP offers the possibility to send a request to this server to calculate the protein stability changes. Since the computation time increases with the number of nsSNPs in the input file, users can decide whether to generate and include this information or just focus on the remaining information.

### Cluster analysis of nsSNPs

Several mutated residues in one protein may have a synergetic effect on the cause and severity of a disease phenotype. The detection of putative quantitative effects requires 3D structural information and visualization. To support the visual inspection of spatial relations, we implemented a hierarchical bottom-up clustering performed on the 3D structure and the included nsSNPs. The applied distance metric refers to the Euclidean distance of the mutated residues’ C-alpha atoms. The linkage criterion to determine the distance between sets of nsSNPs was defined according to the average linkage variant. The results of the cluster analysis are represented in tabular format on BALL-SNP’s information page. Within the 3D structure visualization the clustering nsSNPs can be labeled according to their cluster affiliation.

### Analysis of DCM data with BALL-SNP

To avoid artifacts, that may arise from using artificially generated datasets and to prove the benefits of the developed tool BALL-SNP, we applied it to the high-quality NGS dataset of 639 DCM patients.

There are two practical scenarios based on NGS data, which are, to the best of our knowledge, not implemented in the previously existing methods: the assessment of the effect of several nsSNPs within in a single protein, and the contribution of one or more nsSNPs to ligand binding or protein stability. BALL-SNP is able to support the user in selecting candidate nsSNPs for further analysis and finding possible solutions in both scenarios. Within the underlying cardiomyopathy dataset, we identified three cases exemplary for both of them. Genes *JUP*, *VCL*, and *SMYD2* revealed nsSNP clusters in potentially interesting locations. In particular, the nsSNPs of the DCM data within these genes reveal no pathogenic annotations. The input files for these genes can be downloaded from the BALL-SNP homepage ([26]).

### Contribution of nsSNPs on protein binding

**Fig. 2:**
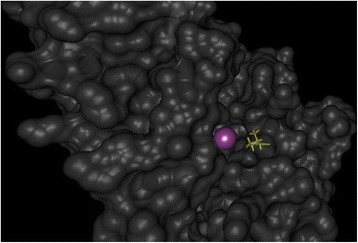
Cutout of the solvent-excluded surface of JUP. The purple sphere represents the center of a predicted binding site. The nsSNP L527I (highlighted in yellow) is located within the detected, putative binding pocket

The gene *JUP* coding for junction plakoglobin is involved in cell junction, which influence the arrangement and function of cells within a tissue. In particular, *JUP* is involved in arrhythmogenic right ventricular dysplasia (ARVD), a congenital heart disease [27]. The nsSNPs in our dataset, identified in the coding region of JUP obtained either no annotation, or were annotated as benign. However, using BALL-SNP, we are able to identify a change in protein stability upon mutation corresponding to several previously unknown nsSNPs. Since the majority of detected nsSNPs within *JUP* have been predicted to induce a decrease in protein stability, they can contribute to dysfunction. Moreover, nsSNP L527I is located within a predicted binding site (Fig. 2). As a consequence, this particular nsSNP can be significantly involved in the observed phenotype due to modification of the protein ligand binding.

### nsSNP cluster

**Fig. 3:**
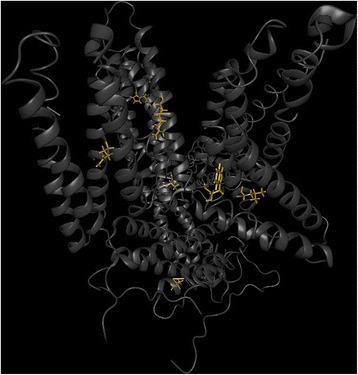
Chain A of protein VCL. The wild type structure of the protein *VCL* is displayed in the so-called cartoon representation (gray). The mutated residues, clustering in the structure, are colored in orange

*VCL* codes for vinculin, an actin filament-binding protein, involved in both, cell-matrix and cell-cell adhesion. *VCL* has been reported to be associated with dilated cardiomyopathy, a congestive heart failure [28]. Database search yields no annotations for the nsSNPs in *VCL* from our dataset. Interestingly, BALL-SNP identifies, that amino acids corresponding to nsSNPs, cluster together in the protein structure (Fig. 3). The nsSNPs R230H, A922V, and H363R, R759Q cluster pairwise with a C-alpha atom distance around 19 Å. In addition, all of these nsSNPs are predicted to decrease protein stability. Interestingly, the nsSNPs I519L, R586W, and V658A cluster with C-alpha atom distances between 15 to 19 Å, and the latter increases protein stability, in contrast to I519L and R586W.

**Fig. 4:**
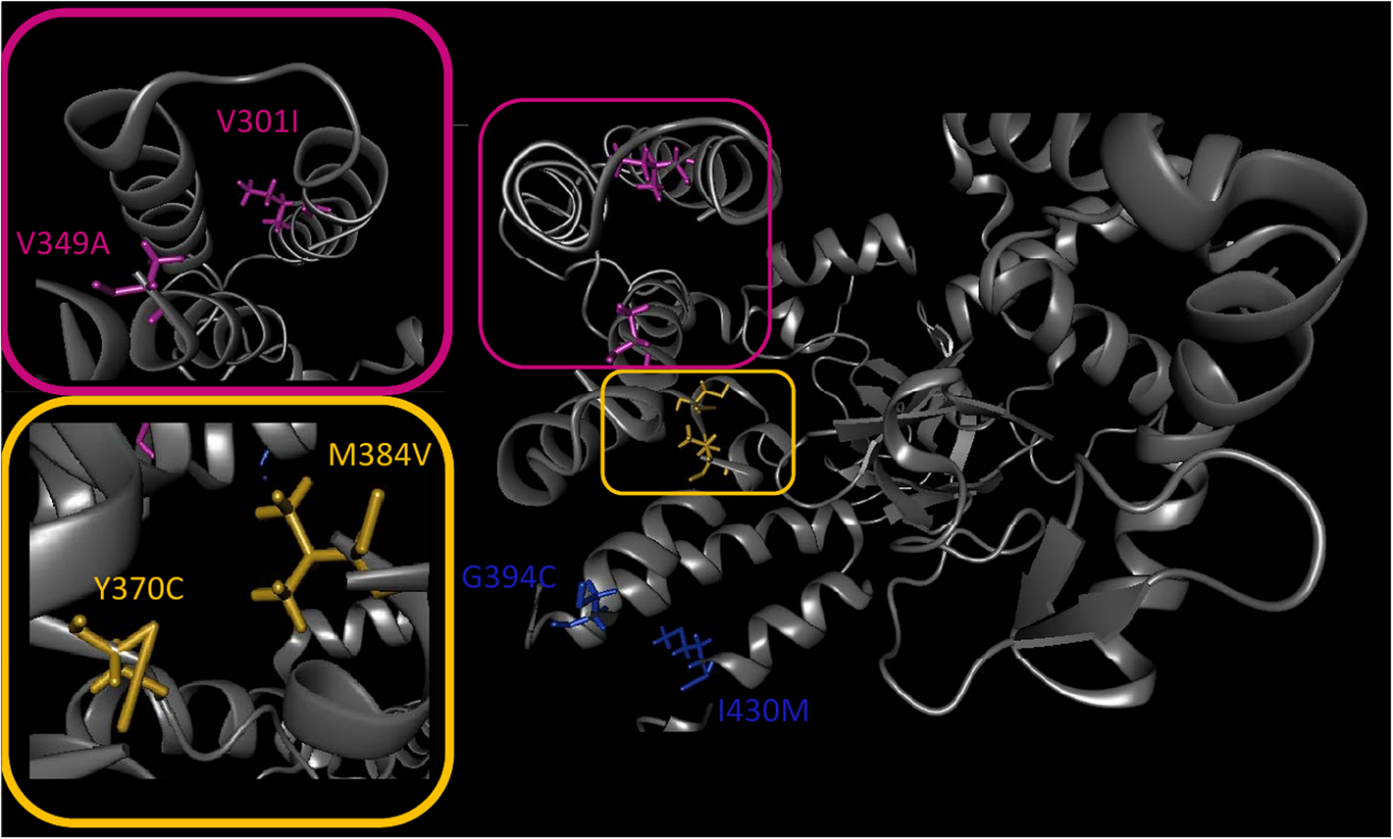
Cutouts of the protein structure of SMYD2. The clustering pairs of nsSNPs are highlighted in different colors. The color framed pictures are close-up views of the correspondingly colored nsSNP pairs. All pairs are located next to each other, indicating a cumulative effect

**Fig. 5:**
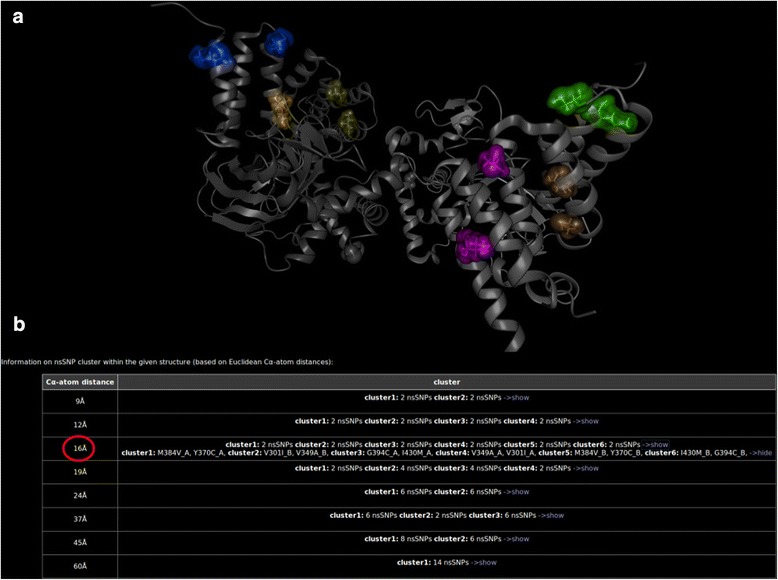
Cluster analysis of the nsSNPs in SMYD2. SMYD2 consists of two chains, (**a**) and (**b**). Hence, the nsSNPs are labeled accordingly. **a** The nsSNPs are highlighted within the protein 3D structure according to their cluster affiliation. **b** The overall cluster analysis results are shown in tabular format on the information page. The red marked distance refers to the highlighted cluster of nsSNPs in the structural visualization

The most prominent example of the cumulative effect of several nsSNPs within our dataset refers to *SMYD2*, coding for a N-lysine methyltransferase, which methylates both, histones and non-histone proteins. While the database search only returns either no or benign annotations, BALL-SNP impressively shows that several nsSNP pairs are located next to each other, implying a cumulative effect. The nsSNPs Y370C and M384V (at a C-alpha atom distance of 9 Å) are adjacent in an opposite direction, and both are predicted to lead to decreased protein stability. Furthermore, the mutations G394C and I430M are located close to each other (12 Å C-alpha atom distance) as well as V301I and V349A (16 Å C-alpha atom distance). Interestingly, both pairs produce opposite predictions concerning their impact on protein stability. Their location next to each other may have a compensating effect on protein stability, since one partner putatively decreases while the other increases it. Figure 4 illustrates these 3D observations, in detail. The overall results of the hierarchical cluster analysis based on average linkage are shown in Fig. 5.

These examples underline the importance of spatial analysis of amino acid substitutions corresponding to nsSNPs within a protein. The presented results certainly require further analysis and experimental validation, however, they reveal the power of BALL-SNP to capture mutual relations and spatial traits of nsSNPs and recover functional features, not available in databases. In medical application, a proper selection of candidate nsSNPs for further analysis can save costs and time. BALL-SNP definitively can support this essential step.

Our tool BALL-SNP combines genetic and structural information to provide scientists the possibility to get deeper insights on the potential effects of accumulated mutations in proteins. We intend to add further functionality in the future development of our tool to overcome some (current) downsides. Often information from databases is not available. Hence, we plan to make use of other existing tools to predict the functional impact of single nsSNPs. Several tools for the pathogenicity prediction of nsSNPs are available. However, the underlying databases and resources exceed the portable size of a downloadable, freely available software tool with a comprehensive molecular modeling library, such as BALL-SNP. Furthermore, the required input formats as well as the obtained output are often incompatible among the tools and thus, a combination of different prediction tools requires additional analysis. We were able to show in a recent study [7] that prediction accuracy and sensitivity can be further improved calculating a sophisticated consensus score for each single nsSNP. We will extend the created compute server with functionality to calculate the prediction results of selected pathogenicity tools as well as a defined consensus score based on the single prediction results.

BALL-SNP currently relies on the 3D information deposited in the PDB. Unfortunately, the gap between known protein sequences and available 3D protein structures is still huge. To solve this problem, we will add the possibility to automatically search for templates and create molecular models for proteins without an available structure in the PDB.

Since BALL enables explicit solvent molecular dynamics (MD) simulations and different docking scenarios, we additionally aim to integrate other functionality and workflows for therapeutic use, such as the analysis of drug target binding.

Since BALL-SNP is an open source project and due to its modular architecture, it is easily extendable and adaptable to include further databases and third party tools, even by other experienced users.

## Conclusion

The analysis of the genotype-phenotype relation and in particular, of the influence of nsSNPs on protein stability and function, is essential in human healthcare. In spite of the fact that the majority of common diseases such as cardiomyopathy are caused by accumulation of several nsSNPs, computational methods to analyze cumulative nsSNPs and their putative quantitative contribution to an observed pathogenic phenotype are missing. In consequence, the validation of the clinical relevance of nsSNP spatial interactions is limited.

Here, we present a novel, freely available software tool, BALL-SNP, which enables the assessment of the impact of nsSNP clusters on protein stability, and consequently assists the selection of candidate nsSNPs for experimental validation. Since both, genetic and structural information is crucial for analysis of the influence of nsSNPs on phenotypes. BALL-SNP is based on a standard molecular modeling framework, allows the use of standard NGS output, and embeds important nsSNP annotation databases.

Though further improvement is needed to meet requirements of the clinical application, BALL-SNP already makes an important contribution to the existing instruments of candidate nsSNP analysis.
